# Electronic structure, elasticity, Debye temperature and anisotropy of cubic WO_3_ from first-principles calculation

**DOI:** 10.1098/rsos.171921

**Published:** 2018-06-20

**Authors:** Xing Liu, Hui-Qing Fan

**Affiliations:** State Key Laboratory of Solidification Processing, School of Materials Science and Engineering, Northwestern Polytechnical University, Xi'an 710072, People's Republic of China

**Keywords:** first principle, electronic structure, elastic properties, Debye temperature, acoustic wave velocity, cubic WO_3_

## Abstract

The electron structure, elastic constant, Debye temperature and anisotropy of elastic wave velocity for cubic WO_3_ are studied using CASTEP based on density functional theory. The optimized structure is consistent with previous work and the band gap is obtained by computing the electronic structure; the top of the valence band is not at the same point as the bottom of the conduction band, which is an indirect band-gap oxide. Electronic properties are studied from the calculation of band structure, densities of states and charge densities. The bulk and shear moduli, Young's modulus, hardness and Poisson's ratio for WO_3_ are studied by the elastic constants. We calculated acoustic wave velocities in different directions and estimated the Debye temperature from the acoustic velocity. The anisotropy of WO_3_ was analysed from the point of view of a pure wave and quasi wave.

## 1. Introduction

**Figure 1.**
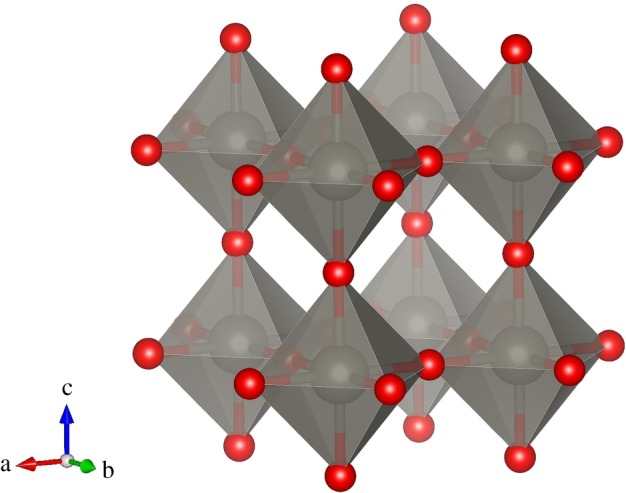
Crystal structure of cubic WO_3_. There are four atoms in the WO_3_ primitive cell: three O atoms at (0, 0, 0), (0, a/2, a/2) and (a/2, 0, a/2) and one W (0, 0, a/2), where a is the lattice constant.

As a type of excellent semiconductor material, tungsten trioxide (WO_3_) has been widely used in multiphase catalysis, electroluminescence, photodegradation, high-temperature superconductivity and new energy fields [1–10]. The ideal cubic WO_3_ primitive cell is a kind of octahedral structure; W and O occupy the central and six corners of the octahedron (figure 1), respectively.

Tungsten trioxide experiences different structure transitions in the temperature range of −180 to 900°C; these phase transitions are not the recombination of tungsten and oxygen atoms, but the distortion of tungsten atoms in the original ideal crystal structure. The phase transition, from low to high temperature; is irreversible; a stable phase that forms at high temperature is also stable at low temperatures. As the temperature increases, the sequence of WO_3_ phase transitions is as follows: monoclinic (at low temperature) → triclinic → monocline (at room temperature) → orthorhombic phase → tetragonal phase. Of course, there is also hexagonal phase WO_3_. Although cubic WO_3_ has not been observed at high temperatures, in many works it is considered as a reference structure, with many reports on experimental and theoretical studies of WO_3_.

**Figure 2.**
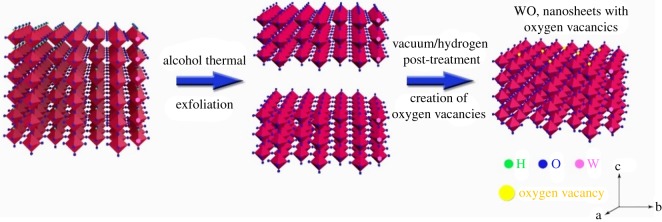
Schematic illustration describing the formation of tungsten oxide single crystal nanosheets [23].

**Figure 3.**
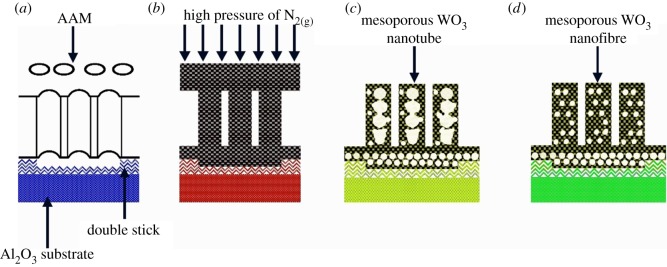
Gas-filled process of mesoporous tungsten oxide nanowire synthesis from triblock co-polymer within a porous anodic alumina membrane (AAM) [25].

Since the 1970s, when Randin [11] found tungsten oxide with photochromic performance for the first time, many research works both at home and abroad have conducted a number of theoretical and experimental studies on photocatalysis, capacitance performance, photosensitivity and gas sensitivity of tungsten oxide [12–21]. Yan *et al*. [22] published an article on hydrogenation of WO_3_. They synthesized tungsten oxide single-crystal nanosheets via the exfoliation of layered tungstic acid to tungsten oxide nanosheets and subsequent introduction of oxygen vacancies (figure 2). In Taiwan, Hsieh *et al*. [23] studied the growth along the [001] direction of triclinic WO_3_ nanowires, and the optical absorption of nanowires by the red shift phenomenon. The results showed the influence of oxygen vacancy on the red shift, and the influence of crystallinity and particle size distribution of the nanowires on the forbidden bandwidth. Hao Lai *et al.* [24] fabricated mesoporous WO_3_ nanofibres and tube-like nanofibres by the gas-filled assistant sol–gel immersion method with porous anodic alumina membrane confinement, which has a porous structure (figure 3). Wang *et al*. [25] studied the structural and electronic properties of WO_3_ by various hybrid functionals combined with both plane wave and localized basis sets. Levy & Pagnier [26], through the establishment of sheet WO_3_ to simulate a nanoscale with the required stoichiometric ratio and oxygen-containing defects, computed the position of the atoms, electron density and the electron state density distribution, and pointed out that the WO_3_ surface could absorb oxygen atoms, which is due to the existence of the acceptor level at the bottom of the surface valence band. Chatten *et al*. [27] studied the WO_3_ electron structure of the oxygen vacancy model in different crystal systems and its forbidden band gap, and found that the polarizations of the bonding and anti-bonding states were not only related to the location of the crystal system but also to the position of oxygen vacancy.

By reviewing the previous work, it has been found that the study of tungsten oxide experimentally and theoretically has made great advancements, but systematic studies of its elastic properties, Debye temperature and anisotropy are rare. It is well known that elastic properties can be used to provide information about the potential of atoms and relate to a variety of basic solid-state phenomena. Therefore, this paper is based on density functional theory (DFT) to study the cubic WO_3_ electronic structure, elastic properties, Debye temperature and anisotropy in different directions.

## 2. Computational details

In recent years, the methods used for theoretical study of metal oxides have been mainly based on LDA, GGA, LDA + U and GGA + U [28–34]; Hubbard parameter U is known as the On-site Coulomb interaction energy [35]. However, a strong association effect is not considered in the calculation of LDA and GGA, that is, the unoccupied d and f orbits. Furthermore, because of the complexity of the electron cloud diffusion, the multibody effect of an orbit is difficult to accurately describe by LDA and GGA. In this system, the energy band structure of the metal is often given by LDA or GGA, and the bands across the Fermi level are often d or f orbits. In fact, the band structure of the system has semiconductor characteristics, there is a clear band gap between the bonding and the anti-bonding states, and the d or f orbit is tightly confined to the nucleus and does not show delocalization. To better describe the strong association system, it is necessary to surpass the traditional LDA or GGA approximation. In this respect, the more successful methods of improvement are LDA + U and GGA + U. Compared with the traditional DFT, the calculation of LDA(GGA) + U does not increase obviously, and the calculation results can be improved significantly with the proper parameters.

**Figure 4.**
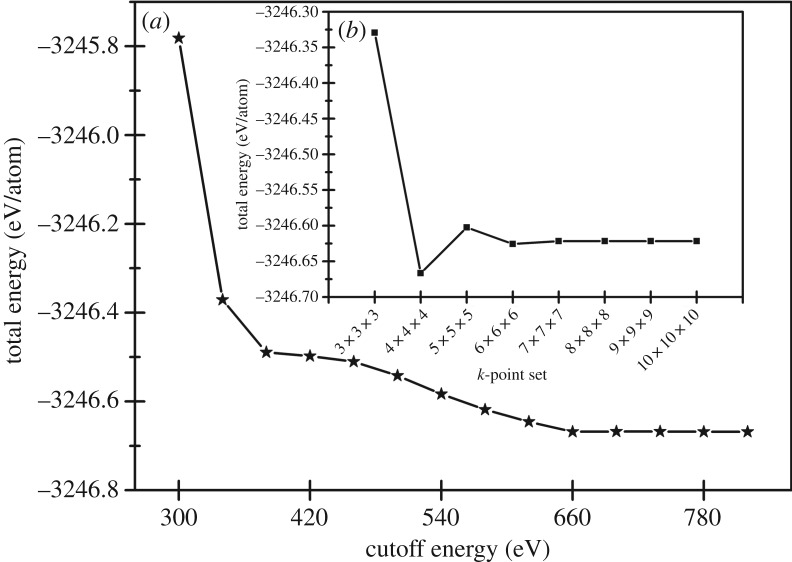
Convergences of the total energy of WO_3_ at different computational parameters. (*a*) Total energy versus the cut-off energy for the *k*-point of 4 × 4 × 4. (*b*) Total energy versus the *k*-point for the cut-off energy of 700 eV.

In this work, we use DFT to study electronic structure, elastic properties, Debye temperature and anisotropy of cubic WO_3_; the calculations have been performed by the CASTEP code [36,37]. By comparison of different approximation methods, LDA + U [38] is finally determined for structural optimization. W 5d^4^6s^2^ electrons and O 2s^2^2p^4^ electrons are explicitly treated as valence electrons. According to the results of the convergence (figure 4), the cut-off energy of the plane wave is taken as *E*_cut_* *= 700 eV, the *k-*point integral in the Brillouin zone is set to 7 × 7 × 7 and the self-consistent convergence method is used to optimize the structure of cubic WO_3_.

## 3. Results and discussion

**Figure 5.**
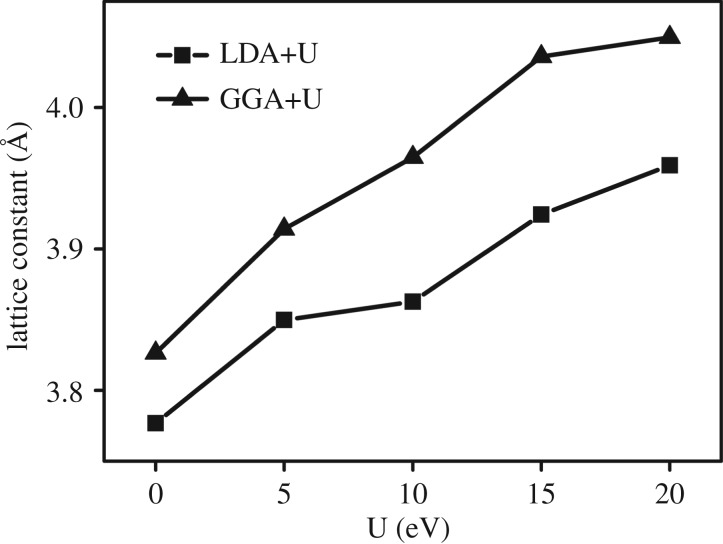
Optimized lattice constant of WO_3_. U values are specified where applicable.

We optimized at the LDA + U (U = 5 eV [35]) level the lattice constant of the cubic WO_3_ to be 3.82 Å, which agrees quite well with those of previous self-consistent calculations: *a* = 3.73–3.84 Å [39–41], as well as the experimental value of *a* = 3.71–3.75 Å [42]. Optimized results by GGA + U are larger than those of the above theoretical and experimental studies (figure 5), which indicates that LDA + U is more suitable for structural optimization than GGA + U in this work.

### 3.1. Electronic structure

The calculated band gap, *E*_g_ = 0.544 eV, is in line with the previous theoretical studies, *E*_g_ = 0.3–0.6 eV [43,44], which is much smaller than the experimental value of 2.62 eV (for the monoclinic phase) [45] due to the well-known DFT error. The results can be modified by using the scissors operator [46], and the band-gap width of WO_3_ is *E*_g_ = 2.70 eV, which does not affect our next step of calculation.

**Figure 6.**
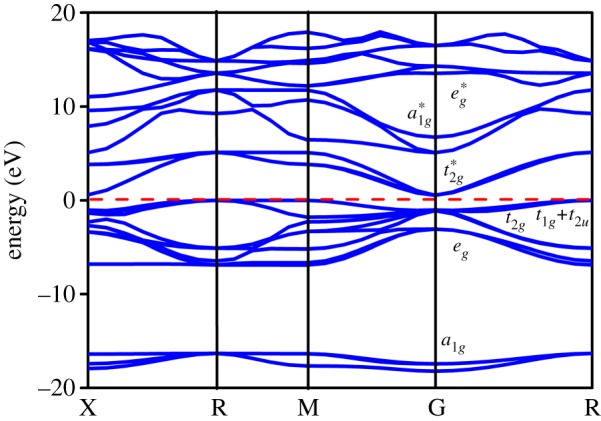
Band structure of WO_3_. The red dashed line represents the Fermi energy level.

As shown in figure 6, the WO_3_ band gap can attain the maximum value at the R point (the top of the valence band) and the minimum value at the G point (bottom of the conduction band), so WO_3_ is an indirect band-gap semiconductor. The valence band consists of 12 levels, which can be divided into four bands: three bonding and one non-bonding bands of hybridized O-2s, -2p and W-5d, -6s states. Based on the crystalline field theory, it can be seen that the lowest three states form $a_{1g}$, which is mainly due to the hybridization of the O-2s orbitals with a few of W 5d(*x^2^–y^2^*) and 5d(*z^2^*). Three states of $e_{g}$ are formed by the hybrids of W-5d(*z^2^*) and 5d(*x^2^–y^2^*) with O-2p orbitals. The energy of $e_{g}$ is lower than that of the $t_{2g}$ band, because the overlap of the d orbitals with O-2p orbitals is stronger than that of the *t_2 g_* band, which is generated by the interaction between W-5d (*d_xy,_ d_xz_, d_yz_*) and O-2p orbitals. Three levels near the top of the valence band form the $t_{1g}+t_{2u}$ non-bonding band; the formations are due to the interaction between the nearest O–O. In the conduction band, the energy of $t_{2g}^{*}$ (anti-bonding band) is lower than that of $e_{g}^{*}$ (anti-bonding band). It is worth noting that the $a_{1g}^{*}$ anti-bonding band is between $t_{2g}^{*}$ and $e_{g}^{*}$.

**Figure 7.**
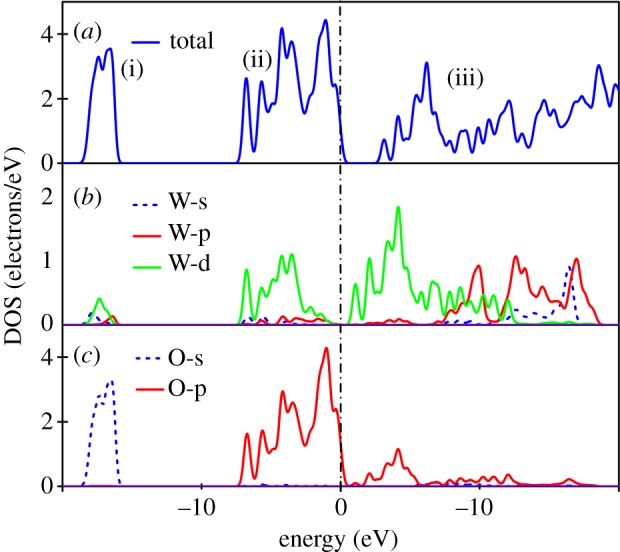
(*a*-*c*) The total and partial density of state for cubic WO_3._

**Figure 8.**
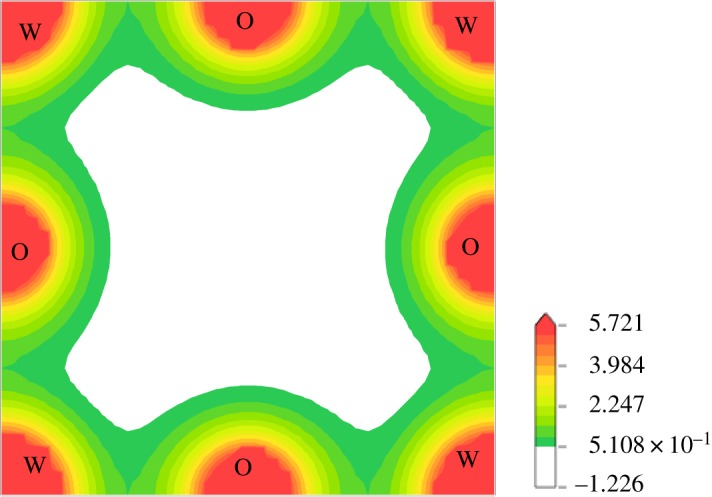
Charge densities of cubic WO_3._

As can be seen in figure 7, the calculated density of states (DOS) of WO_3_ is divided into three groups. The low-valence band (i), that is, with a bandwidth of 2.93 eV, is mainly composed of O-2s, and a minority of W-5d. Group (ii), near the top of the valence band, with a bandwidth of 7.63 eV, is formed by hybridization between O-2p and W-5d, and the contribution of tiny amounts of W-5p, -6 s. Group (iii) is located in the conduction band with a bandwidth of 13.87 eV. In the bottom of the conduction band, we found that there is a mixture of dominant O-2p with W-5d $t_{2g}^{*}$ orbitals, and in the upper area of group (iii), the main contribution is by hybridization between W-5p and -6s electrons. The electronegativity for W (1.7) and O (3.44) has a small difference; consequently, hybridized peaks are dispersive and intensities are not strong enough, which is also the cause of WO_3_ instability. Furthermore, the total charge densities of cubic WO_3_ is presented in figure 8; it shows that the bonds between W and O are covalent due to hybridization, which agrees well with our analysis of DOS.

### 3.2. Elastic and mechanical properties

According to Hooke's law, there are only three independent elastic constants *C*_11_, *C*_12_ and *C*_44_ for cubic crystal structures (*C*_11_ = *C*_22_ *=* *C*_33_, *C*_12_ *=* *C*_13_ *=* *C*_23_, *C*_44_ *=* *C*_55_ *=* *C*_66_). Here. *C*_11_ = 546, *C*_12_ = 35 and *C*_44_ = 71. Based on the Born's stability restrictions [47]:

3.1 $$C_{11}>0,\;C_{44}>0,\;C_{11}>|C_{12}|,\;(C_{11}+2C_{12})>0.$$

It is known that the elastic constants of the cubic WO_3_ are satisfied with the above stability conditions, so the cubic WO_3_ is stable in mechanics.

**Figure 9.**
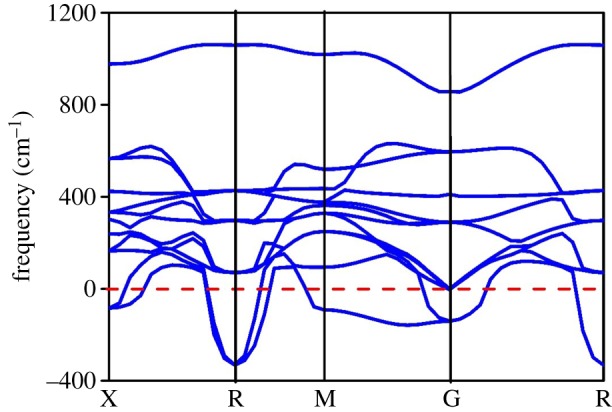
Phonon dispersion diagram for WO_3_.

We further calculated the phonon dispersion of WO_3_, whose imaginary frequency can be found from figure 9, which indicates that the cubic WO_3_ structure is unstable, which is not a contradiction with the above equation (3.1). In cubic WO_3_, the obtained results by the Born's stability restrictions show that the stability is affected by the external force, that is, from the point of view of macroscopic mechanics. While the imaginary frequency of the phonon dispersion shows that the atomic arrangement is unstable at present, from the point of view of microscopic atomic structure, atoms in the WO_3_ will be rearranged by the lattice vibration to form a new and stable structure.

The bulk and shear modulus are two key parameters for the characterization of material hardness. The bulk modulus can be described as the ability of the material to resist the change of bulk, and is also understood as the mean value of the bond strength. The shear modulus can be described as the ability of the material to resist the shape change caused by shear force, and the ability to resist the change of the bond angle. The calculation of the bulk and shear modulus can be obtained by *C_ij_*, and there are two different methods to do so. One is the calculation of the strain continuity on grain boundaries by Reuss [48], and another is Voigt's [49] proposed stress continuity on the grain boundary. Hill [50] proves that the calculations of the Reuss and Voigt models are the lower and upper limit of the elastic constants, respectively, so the Hill model calculates the arithmetic mean of the results of the Reuss and the Voigt models:

3.2 $$B_{\mathrm{Hill}}=\frac{1}{2}(B_{\mathrm{Reuss}}+B_{\mathrm{Voigt}});\;G_{\mathrm{Hill}}=\frac{1}{2}(G_{\mathrm{Reuss}}+G_{\mathrm{Voigt}}),$$

where

3.3 $$\begin{array}{rl} B_{R} & =B_{V}=\frac{1}{3}(C_{11}+2C_{12}), \end{array}$$

3.4 $$\begin{array}{rl} G_{V} & =\frac{1}{5}(C_{11}-C_{12}+3C_{44}) \end{array}$$

3.5 $$\begin{array}{rl} \;\text{and}\;G_{R} & =\frac{5(C_{11}-C_{12})C_{44}}{4C_{44}+3(C_{11}-C_{12})}. \end{array}$$

The Young's modulus *E* of cubic crystal material can be expressed as the corresponding coefficient:

3.6 $$E=\frac{9B_{x}G_{x}}{G_{x}+3B_{x}},$$

where *x* represents Reuss, Voigt and Hill.

The comparison of our calculated data and available theoretical studies [51–54] has been presented in table 1. It is obvious that our calculation constants are in accordance with previous work. According to the criterion of Pugh [55], *B*_X_/*G*_X_, which is defined as the shear modulus corresponding to the plastic deformation, and the bulk modulus are related to the fracture resistance. Pugh proposed that if *B*_X_/*G*_X_ exceeds the critical value (1.75), the material will have ductility. In our calculation, the value is less than 1.75, which indicates that cubic WO_3_ has little ductility, which is also explained in the next study on Poisson's ratio.

**Table 1.** According to equations (3.2)–(3.6), the bulk modulus, shear modulus and Young's modulus of WO_3_ are calculated.

bulk modulus			shear modulus		
*B*_V_	*B*_R_	*B*_H_	*G*_V_	*G*_R_	*G*_H_
205	205	205	145	100	123
228(DFT), 225(ABOP) [51]; 224(DFT) [44]; 151 [52]; 254 [53]			123(DFT) [55]		

Young's modulus			Pugh		
*E*_V_	*E*_R_	*E*_H_	*B*_V_/*G*_V_	*B*_R_/*G*_R_	*B*_H_/*G*_H_
352	258	305	1.41	2.05	1.67
311(DFT) [54]					

Hence, we introduced the study of the Vickers hardness, which is an empirical formula for evaluating the hardness of the material.

3.7 $$H_{V}=2{\left(k^{2}G\right)}^{0.585}-3$$

and

3.8 $$\upsilon =\frac{1}{2}\frac{B_{x}-(2/3)G_{x}}{B_{x}+(1/3)G_{x}},$$

where *k* is the Pugh ratio, which is the ratio of the shear *G* to the bulk *B* modulus, *k = G/B*.

Compared to other metal oxides in table 2, we can see that WO_3_ has greater hardness. This is due to the fact that the bulk modulus is not directly related to the hardness, and the shear modulus is more capable of characterizing the hardness of the material. Furthermore, with regard to the Poisson ratio, it reflects the strength of the covalent bond in the material. In general, the Poisson ratio 0.1 ∼ 0.28 represents the covalent property of the material, while 0.29 ∼ 0.33 represents the metal characteristics. The Poisson ratios for the above metal oxides, except for WO_3_ and MgO, are more than 0.29, which means that they have metal properties along with their mechanical properties, and their hardness is less than that of WO_3_.

**Table 2.** Vickers’ hardness and Poisson's ratio for WO_3_ compared to the other calculated results.

	*B*	*G*	H_V_	*υ*
WO_3_	205	123	15.3	0.252
ZrO_2_ [56]	196	92	8.62	0.297
HfO_2_ [57]	293	103	5.85	0.342
TiO_2_ [58]	174	70	5.27	0.363
CaO [59]	144	88	12.4	0.266
MgO [60]	139	114	22.3	0.178

### 3.3. Debye temperature

Debye temperature is a very important thermodynamic parameter reflecting thermodynamic properties, which is related to many physical properties of solids, such as acoustic velocity, specific heat capacity and thermal expansion coefficient. According to the above bulk and shear modulus, we calculated wave velocity *v_s_*, longitudinal wave velocity *v_p_* and average wave velocity *v_m_*, and further studied the Debye temperature of WO_3_ by equations (3.9) and (3.10). The relationship between the Debye temperature and the average wave velocity is as follows:

3.9 $$v_{s}=\sqrt{\frac{G_{x}}{\rho }};v_{p}=\sqrt{\frac{B_{x}+4G_{x}/3}{\rho }};v_{m}={\left(\frac{2/{\upsilon_{s}}^{3}+1/{\upsilon_{p}}^{3}}{3}\right)}^{-1/3}$$

and

3.10 $$\Theta =\frac{h}{k}{\left(\frac{3nN_{A}\rho }{4\pi M}\right)}^{1/3}v_{m},$$

where *h* is the Planck constant, *k* is the Boltzmann constant, *N*_A_ is the Avogadro constant, *M* is the molecular weight and *ρ* is the density (table 3).

**Table 3.** Calculation of the elastic shear wave velocity *v*_s_, longitudinal wave velocity *v*_p_, average wave velocity *v*_m_ and the Debye temperature of WO_3_.

$v_{s}^{X}$			$v_{p}^{X}$		
*X* = V	*X* = R	*X* = H	*X* = V	*X* = R	*X* = H
4659	3869	4291	7722	7117	7432

$v_{m}^{X}$			*Θ*^X^		
*X* = V	*X* = R	*X* = H	*X* = V	*X* = R	*X* = H
5151	4316	4763	630	533	582

The Debye temperature is calculated to be 582 K; unfortunately, there are no data available for comparison in the literature, as far as we know. To analyse the results, we only compared other functional materials, such as GaN (390), ZnO (303) and Al_2_O_3_(370) [61,62]; it can be found that WO_3_ has a higher Debye temperature, which shows that the interatomic binding force, melting point, hardness and thermal expansion coefficient of WO_3_ are larger than those of the above materials.

### 3.4. Anisotropy of elastic wave velocity

For cubic WO_3_, the propagation velocity of longitudinal and transverse waves along different crystal directions is related to the elastic constants *C_ij_*. In table 4, the equations relating velocities of propagation and elastic constants used for the calculations on WO_3_ are given.

**Table 4.** Relation of velocity to elastic constants for various modes of propagation.

orientation of WO_3_ sample	mode of propagation	relation of velocity to elastic constants	calculated velocity
[100]	longitudinal	$v_{1}=\sqrt{C_{11}/\rho }$	9040
[100]	transverse	$v_{2}=\sqrt{C_{44}/\rho }$	3260
[110]	longitudinal	$v_{3}=\sqrt{(C_{11}+C_{12}+2C_{44})/2\rho }$	7356
[110]	transverse	$v_{4}=\sqrt{C_{44}/\rho }$	3260
[110]	transverse	$v_{5}=\sqrt{(C_{11}-C_{12})/2\rho }$	6184
[111]	longitudinal	$v_{6}=\sqrt{(C_{11}+2C_{12}+4C_{44})/3\rho }$	6701
[111]	transverse	$v_{7}=\sqrt{(C_{11}-C_{12}+C_{44})/3\rho }$	5389

From table 4, we can draw a conclusion: for cubic WO_3_, *v*_1_ is the largest of longitudinal wave velocities; the wave is a pure longitudinal wave in the direction of [100], while in [110] and [111], waves of *v*_3_ and *v*_6_ are quasi-longitudinal waves. In a pure longitudinal wave, the direction of the particle vibration is perpendicular to the forward direction of the wave, whereas for the quasi-longitudinal wave it is not so, and the velocity component will be produced in other directions, and therefore *v*_1_> *v*_3_, *v*_6_. The velocities for *v*_3_, *v*_4_ and *v*_5_ are all different; transverse waves, which are formed by the degeneracy split into *v*_4_ and *v*_5_, have different velocities. Based on the above-mentioned reasons, we can conclude that it is anisotropy in this direction.

## 4. Conclusion

In this paper, we have performed first-principles calculations on cubic WO_3_, including structural parameters as well as the band structure, density of state, elastic constants, Debye temperature and the acoustic wave velocity in different directions. The calculated results show that the cubic WO_3_ is an indirect band-gap oxide; the valence band is mainly composed of O-2p, and the bottom of the conduction band is mainly contributed by W-5d and a few of O-2p. There is no doubt about the importance of elastic constants in this work; based on the elastic constants, the bulk, shear and Young's modulus are also further studied, which shows that the shear modulus is determined according to the stability of cubic WO_3_. Vickers' hardness and Poisson ratio are also investigated by obtaining the bulk and the shear modulus; there is no research on the hardness of WO_3_, and we can only compare it with other oxides.

The calculation of Debye temperature is further obtained by elastic constants, as 582 K, which is useful not only for the potential application of WO_3_ on thermoelectric and thermal resistance materials but also for the development of thermoelectric materials in future. Finally, we investigated the acoustic wave velocities in different directions. Based on the concept of pure and quasi wave, and the calculated result of wave velocity, it can be shown that cubic WO_3_ acoustic waves have anisotropy in the [110] direction.

## Supplementary Material

WO3.cif

## Data accessibility

The datasets supporting this article have been uploaded as part of the electronic supplementary material.
